# Alternative splicing analysis benchmark with DICAST

**DOI:** 10.1093/nargab/lqad044

**Published:** 2023-05-30

**Authors:** Amit Fenn, Olga Tsoy, Tim Faro, Fanny L M Rößler, Alexander Dietrich, Johannes Kersting, Zakaria Louadi, Chit Tong Lio, Uwe Völker, Jan Baumbach, Tim Kacprowski, Markus List

**Affiliations:** Chair of Experimental Bioinformatics, Technical University of Munich, 85354 Freising, Germany; Institute for Computational Systems Biology, University of Hamburg, Notkestrasse 9, 22607 Hamburg, Germany; Institute for Computational Systems Biology, University of Hamburg, Notkestrasse 9, 22607 Hamburg, Germany; Chair of Experimental Bioinformatics, Technical University of Munich, 85354 Freising, Germany; Chair of Experimental Bioinformatics, Technical University of Munich, 85354 Freising, Germany; Chair of Experimental Bioinformatics, Technical University of Munich, 85354 Freising, Germany; Chair of Experimental Bioinformatics, Technical University of Munich, 85354 Freising, Germany; Chair of Experimental Bioinformatics, Technical University of Munich, 85354 Freising, Germany; Institute for Computational Systems Biology, University of Hamburg, Notkestrasse 9, 22607 Hamburg, Germany; Chair of Experimental Bioinformatics, Technical University of Munich, 85354 Freising, Germany; Institute for Computational Systems Biology, University of Hamburg, Notkestrasse 9, 22607 Hamburg, Germany; Interfaculty Institute for Genetics and Functional Genomics, University Medicine Greifswald, Felix-Hausdorff-Straße 8, D-17475 Greifswald, Germany; DZHK (German Centre for Cardiovascular Research), Partner Site Greifswald, Greifswald, Germany; Institute for Computational Systems Biology, University of Hamburg, Notkestrasse 9, 22607 Hamburg, Germany; Institute of Mathematics and Computer Science, University of Southern Denmark, Campusvej 55, 5000 Odense, Denmark; Division Data Science in Biomedicine, Peter L. Reichertz Institute for Medical Informatics of Technische Universität Braunschweig and Hannover Medical School, Braunschweig, Germany; Braunschweig Integrated Centre of Systems Biology (BRICS), TU Braunschweig, Braunschweig, Germany; Chair of Experimental Bioinformatics, Technical University of Munich, 85354 Freising, Germany

## Abstract

Alternative splicing is a major contributor to transcriptome and proteome diversity in health and disease. A plethora of tools have been developed for studying alternative splicing in RNA-seq data. Previous benchmarks focused on isoform quantification and mapping. They neglected event detection tools, which arguably provide the most detailed insights into the alternative splicing process. DICAST offers a modular and extensible framework for analysing alternative splicing integrating eleven splice-aware mapping and eight event detection tools. We benchmark all tools extensively on simulated as well as whole blood RNA-seq data. STAR and HISAT2 demonstrated the best balance between performance and run time. The performance of event detection tools varies widely with no tool outperforming all others. DICAST allows researchers to employ a consensus approach to consider the most successful tools jointly for robust event detection. Furthermore, we propose the first reporting standard to unify existing formats and to guide future tool development.

## INTRODUCTION

Alternative splicing (AS) affects around 95% of eukaryotic genes with multiple exons (1,2) and gives rise to a large number of isoforms. AS is involved in cellular processes and disease development (see recent reviews on cancer (3), muscle (4) and neuron (5) development). The most popular technology to study AS is short-read RNA sequencing (RNA-Seq). Possibilities for AS analysis from short-read RNA-Seq data comprise splice-aware mapping, *de novo* transcriptome assembly, AS detection and/or quantification on the exon, isoform, or event level as well as differential splicing analysis. Each year, several new tools are published for each of these analysis types.

Existing benchmark studies that could guide users on which tool to use for which analysis have several limitations (6–16). First, the studies use as ground truth either only a small subset of well-studied genes or simulated RNA-Seq data with randomly introduced AS events and limited event types. Second, they focused on AS detection tools that operate on the isoform level (6–11), splice-aware mapping tools (12,13), and differential splicing analysis tools (14–16). However, AS detection tools that operate on the event level are more precise. Finally, benchmark studies are seldomly updated.

To perform a standardised benchmark, we created a modular pipeline called DICAST (Docker Integration for Comparison of AS tools). It uses Docker to handle the installation process and Snakemake to handle AS analysis and the benchmark workflow. Its modularity allows for adding new tools in the future and for quickly updating the benchmark. As ground truth, we used RNA-Seq data sets that were simulated genome-wide and contained a predefined number and distribution of AS events. The simulation with ASimulatoR (17) allowed us to evaluate the challenges and limitations of the tested tools systematically across data sets with varying levels of difficulty.

While our main focus was to benchmark AS event detection tools, we started from splice-aware mapping to provide the best possible input to those tools. Thus, we evaluated 11 splice-aware mapping tools in addition to eight AS detection tools. We demonstrate the benefits and limitations of AS event detection tools and suggest a putatively optimal strategy for comprehensive AS analysis.

## MATERIALS AND METHODS

### DICAST

DICAST uses Docker for full reproducibility and to simplify deployment (18,19). Each docker image in DICAST can be used individually or as part of the benchmark/AS analysis pipeline. DICAST orchestrates the pipeline using Snakemake (20). The DICAST source code and documentation are available at https://github.com/CGAT-Group/DICAST and https://dicast.readthedocs.io/en/master/contents.html, respectively.

### Benchmark workflow

Supplementary Figure S1 illustrates the workflow for benchmarking AS analysis tools. RNA-Seq data sets were simulated using the R package ASimulatoR (17) with a sequencing depth of 200 million reads (read length 76 bp) based on the human genome hg38 and the Ensembl genome annotation, version 99 (21). We limited the simulation to chromosomes 1–22, X, Y and MT.

We tuned the complexity of the simulated data sets changing AS events distribution, combinations, and sequencing error rate (Table 1). We started from the data set S0 with equal proportions of the four main types of AS events and 0% sequencing error rate. Using this data set, we filtered out tools that demonstrate poor performance or long run time. Next, we added MES, ALE, and AFE to simulate data set S1 and to evaluate the impact of complex AS events on tool performance. For data set S2, we raised the sequencing error rate up to 0.1% and investigated the impact of not-perfect sequencing. Data sets S0–S2 contain only one event per transcript which is an artificial setting helping us to explore performance under simpler and well-controlled conditions. As all AS event types combinations are possible, we evaluated how the AS event types combinations impact the performance: for data set S3 we allowed two types of events to occur within one transcript but with different exons; for data set S4 we allowed two types of events to occur with the same exon. We also aimed to evaluate AS analysis tools in a biologically relevant setting but the real RNA-Seq data sets lack the genome-wide ground truth. We addressed this challenge and simulated a data set S5 with realistic event proportions, which were obtained by analyzing 117 RNA-Seq samples from the SHIP cohort (the Study of Health in Pomerania) (22).

**Table 1.** Characteristics of the simulated data sets. ‘Transcripts per gene’ indicates the number of alternative transcript variants simulated for a gene; ‘Events per transcript’ indicates the number of AS events simulated within one transcript; ‘Events per exons’ indicates the number of AS events which an exon can be simultaneously involved in (e.g., exon skipping and alternative splice site)

				Event types							
Simulated data set	Transcripts per gene	Events per transcript	Events per exon	ES	IR	A5	A3	MES	ALE	AFE	Sequencing error rate
S0	1	1	1	x	x	x	x				0%
S1	1	1	1	x	x	x	x	x	x	x	0%
S2	1	1	1	x	x	x	x	x	x	x	0.1%
S3	>=1	2	1	x	x	x	x	x	x	x	0.1%
S4	>=1	2	>=1	x	x	x	x	x	x	x	0.1%
S5	>=1	2	>=1	x	x	x	x				0.1%

For each simulated data set, ASimulatoR estimated how many genes have enough exons to create AS events and can, hence, be used for the simulation. For the simulated set S3, this number was the lowest—37 648. We used this number of genes as a parameter for all simulated data sets to preserve the read coverage level.

We used seqtk (available at https://github.com/lh3/seqtk) to downsample fastq files uniformly at random to 10 million reads to benchmark splicing-aware mapping tools. We then mapped these data sets to the human genome using 11 splice-aware mapping tools and calculated the proportion of unmapped reads relative to the number of all simulated reads (‘fraction of unmapped reads’) and the proportion of correctly mapped reads and junctions relative to the number of all mapped reads (‘precision’). Precision and fraction of unmapped reads for splice-aware mapping tools were calculated based on the resulting alignment file and the correct coordinates provided by ASimulatoR.

We chose the mapping tool with the best balance between precision and fraction of unmapped reads values and used it to produce alignments for 8 AS detection tools. We analyzed fastq files with 200 million reads and additionaly used seqtk to downsample them to 50 and 100 million reads. We then calculated the proportion of correctly found events relative to the number of events in the simulated set (‘recall’) and relative to the number of events found by the tool (‘precision’). Precision and recall for AS event detection tools were calculated based on the unified output of DICAST and the description of correct events provided by ASimulatoR.

We estimated the variability in performance of splicing-aware mapping tools and AS detection tools by simulating five samples for the most simple data set (S0) and five samples for the biologically-relevant data set (S5).

### SHIP cohort

The Study of Health in Pomerania (SHIP) is a longitudinal population-based cohort study located in the area of West Pomerania (Northeast Germany). For RNA-Seq analysis, total RNA was extracted from whole blood with a mean RNA integrity of 8.5. Based on 500ng total RNA per sample, a library was prepared using the TruSeq Stranded mRNA kits (A and B) with 24 barcodes and 117 samples were sequenced on Illumina HiSeq 4000, 2× 101 bp paired-end reads with a sequencing depth 40 million clusters per sample. Written informed consent was obtained from SHIP-TREND study participants, and all protocols were approved by the institutional ethical review committee in adherence with the Declaration of Helsinki. We used these 117 RNA-Seq samples from the SHIP-TREND cohort (22) in this study. They were mapped to the human genome hg38 with STAR using the Ensembl genome annotation, version 99, and analyzed further with MAJIQ (23). Custom scripts in Python 3 were used to obtain the number of AS events and genes with AS.

## RESULTS

### Tools for benchmark

We collected splice-aware mapping and AS event detection tools released between 2010 and 2021 based on a Pubmed and Google Scholar search with the following inclusion criteria. A tool should be (i) available, (ii) documented, (iii) under an open-source license, (iv) available as stand-alone software (web-services can usually not process a large amount of data), (v) used in project(s) other than described in the tool publication, (vi) able to process widely used file formats such as fasta/fastq, gtf/gff3, and bam/sam. Since we controlled the number of AS events in a custom genome annotation, a tool should be (vii) able to work with those as well. The final list of tools contains 11 splice-aware mapping tools and 8 AS detection tools (Table 2).

**Table 2.** The list of AS analysis tools chosen for benchmark

Name (version)	Dependencies	Reference	Genome Annotation*
**A. Splice-aware mapping tools**			
BBMap (38.94)	Java 7+	(24)	-
ContextMap2 (2.7.9)	Java, BWA, bowtie 1, bowtie2	(26)	+
In the current study used with bowtie 2 (25)			
CRAC (2.5.2)	Perl, htslib	(27)	-
DART (1.4.6)	GCC, GNU make, libboost-all-dev,	(28)	-
	libbz2-dev, and liblzma-dev		
GSNAP (2020-03-12)	GCC,GNU make, Perl	(29)	+
HISAT2 (2.2.1)	GCC, GNU make, MSYS, zlib	(30)	+
MapSplice2 (2.2.1)	GCC 4.3.3+, GNU make, python 2+	(31)	+
minimap2 (2.17)	None (precompiled binaries) or	(32)	-
	GCC, GNU make, zlib		
segemehl (0.3.4)	GCC, GNU make, htslib	(33)	-
STAR (2.7.5)	GCC, GNU make	(34)	+
Subjunc (2.0.0)	None (precompiled binaries) or	(35)	+
	GCC, GNU make		
**B. Alternative splicing event detection tools**			
Name (version)	Dependencies	Reference	Supported events
ASGAL (1.1.6)	python3.6+, biopython, pysam, gffutils,	(36)	ES, IR, A5, A3
	pandas, cmake, samtools, zlib		
ASpli (1.12.0)	R, BiocManager	(37)	ES, IR, A5 A3
EventPointer (2.4.0)	R, BiocManager	(38)	ES, IR, A5, A3, MEE, MES
IRFinder (1.3.1)	GLIBC 2.14+, GCC 4.9.0+, Perl 5+,		
	STAR 2.4.0+, samtools 1.4+, bedtools 2.4+	(39)	IR
MAJIQ (2.3)	htsilb, python3, python packages	(23)	ES, IR, A5, A3
SGSeq (1.24.0)	R, BiocManager	(40)	ES, IR, A5, A3SS, AFE,
			ALE, AF, AL,
			MES (with two skipped exons)
splAdder (2.4.3)	python3, python packages	(41)	ES, IR, A5, A3, MEE, MES
Whippet (0.11.1)	julia	(42)	New junctions could be
			added from alignment as an option
			ES, A3SS, A5SS, IR, AFE, ALE,
			tandem transcription start, tandem
			alternative polyadenylation, circular
			back splicing

* ‘-’: does not use; ‘+’ - can use as an option

ES, exon skipping; IR, intron retention; A3, alternative 3′-splice site, A5, alternative 5′-splice site, MES, multiple exon skipping, MEE, mutually exclusive exons, AFE, alternative first exon, ALE, alternative last exon.

### Alternative splicing analysis and benchmark pipeline

#### A unified output format for AS event detection

The main challenge for comparing AS event detection tools is the lack of standard output format. For an exon skipping event, ASGAL reports the coordinates of neighboring exons; ASpli reports the coordinates of a skipped exon itself; MAJIQ reports the coordinates of a neighboring exon and its junctions. We propose a unified format for all AS event types that reports the coordinates of (i) skipped exons for exon skipping, multiple exon skipping, mutually exclusive exons; (ii) a retained intron for intron retention; (iii) an alternative part of an exon for alternative splice sites (Supplementary Figure S2).

Additionally, for each event, the unified output format contains the gene name, chromosome, strand as well as a unique ID.

#### DICAST: Docker Integrated Comparison of Alternative Splicing Tools

The next necessity for the standardization of the AS analysis and benchmark process is a unified pipeline. DICAST handles (i) the installation process for every tool using Docker; (ii) the execution of tools using Snakemake; (iii) the output format unification; (iv) the comparison of AS events detected by different tools.

**Figure 1.**
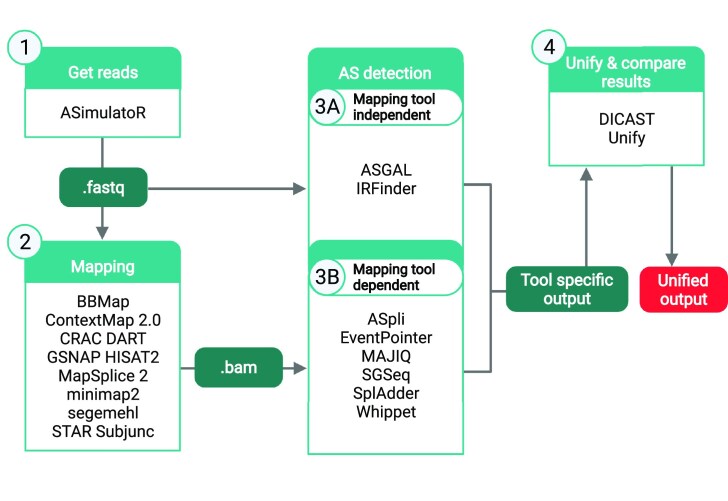
DICAST framework: 1) simulated (ASimulatoR) or user-provided fastq files 2) bam files could be generated by any of 11 supported splice-aware mapping tools; 3) AS events detected by any of 8 AS event detection tools based on files generated in the previous steps; 4) the output files of the AS detection tools are unified by DICAST. Created with BioRender.com.

The general workflow starts with short-read RNA-Seq data (optionally simulated by ASimulatoR (17), Figure 1) that serves as input for splice-aware mapping tools. The resulting alignment files then serve as input for AS event detection tools. Next, DICAST converts the output files of the AS event detection tools to the unified format described above, compares detected events across the tools, and reports the results of the comparison. A user could run all or separate steps of the workflow with their RNA-Seq reads, alignment and genome annotation files.

### Benchmark results: splice-aware mapping tools

Splice-aware mapping tools differ in the mapping approach and their use of genome annotations. Most tools use variations of the seed-and-extend algorithm and start with aligning parts of a read (seeds). ContextMap and MapSplice2 first align reads end-to-end and use the seed-and-extend algorithm for reads that could not be aligned in the first step. Some tools can use genome annotations for additional information (e.g., splice sites) (Table 2), while others (e.g. BBmap) do not need it.

We provided a genome annotation where possible and used the best mode for AS analysis (e.g. a 2-pass mode for STAR). We downsampled the input files to 10 million reads uniformly at random to reduce the analysis time. For data sets S0 and S5 we repeated the alignment with five simulated datastets to evaluate the robustness of the performance.

**Figure 2.**
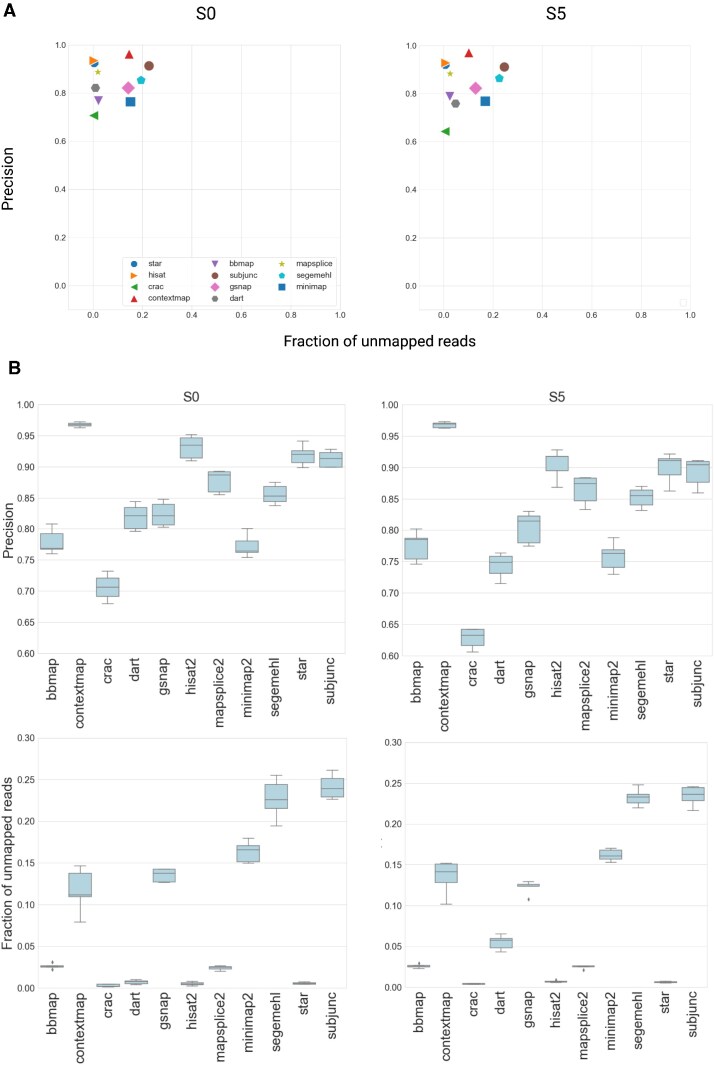
(**A**) The plots for precision and fraction of unmapped reads plots for splice-aware mapping tools for the data sets S0 and S5. The results for S1–S4 are presented in Supplementary Figure S3. The values are taken from the analysis of one out of five simulated datasets as an example. (**B**) The boxplots for precision and fraction of unmapped reads for splicing-aware mapping tools calculated for S0 (repeated 5 times) and S5 (repeated 5 times)

The mapping approach has a limited effect on the performance of the tool. ContextMap and MapSplice2 show comparable performance metrics as the tools with the best performance such as STAR and HISAT2 (Figure 2, Supplementary Figure S3). Tools that use genome annotation generally show better performance. Surprisingly, the complexity of the data sets only marginally affects the performance of the tools. Only DART suffers from a decrease in precision with an increasing sequencing error rate. Splice-aware mapping tools are robust in terms of precision (Figure 2B): the standard deviation is less than 0.02. Concerning the fraction of unmapped reads we observe two categories: BBMap, CRAC, DART, HISAT2 and STAR perform well with a low fraction of unmapped reads and a low standard deviation while others perform poorly in both metrics.

In summary, STAR and HISAT2 showed a balance between precision and fraction of unmapped reads compared to other tools. ContextMap demonstrated the best precision and could be considered for the analysis if the sequencing depth of samples is deep enough and a user can allow the loss of 10–15% of reads. Unfortunately, ContextMap has long run time (see run time subsection below) which might be inconvenient when analyzing a large number of samples. We chose STAR for further analysis, as it is more widely used (28 433 Google Scholar citations by January 2023). We downsampled data sets to 200, 100 and 50 million reads uniformly at random to investigate the effect of sequencing depth on performance.

### Alternative splicing detection tools

AS event detection tools detect events from genome annotation (ASpli, SGSeq (annotated transcripts), IRFinder), RNA-Seq alignment (EventPointer, SGSeq (*de novo*), MAJIQ), or both, i.e. by augmenting the genome annotation through alignment (ASGAL, splAdder, Whippet). As most tools (Table 2) do not detect mutually exclusive exons and multiple exon skipping, we treated such events as exon skipping events.

**Figure 3.**
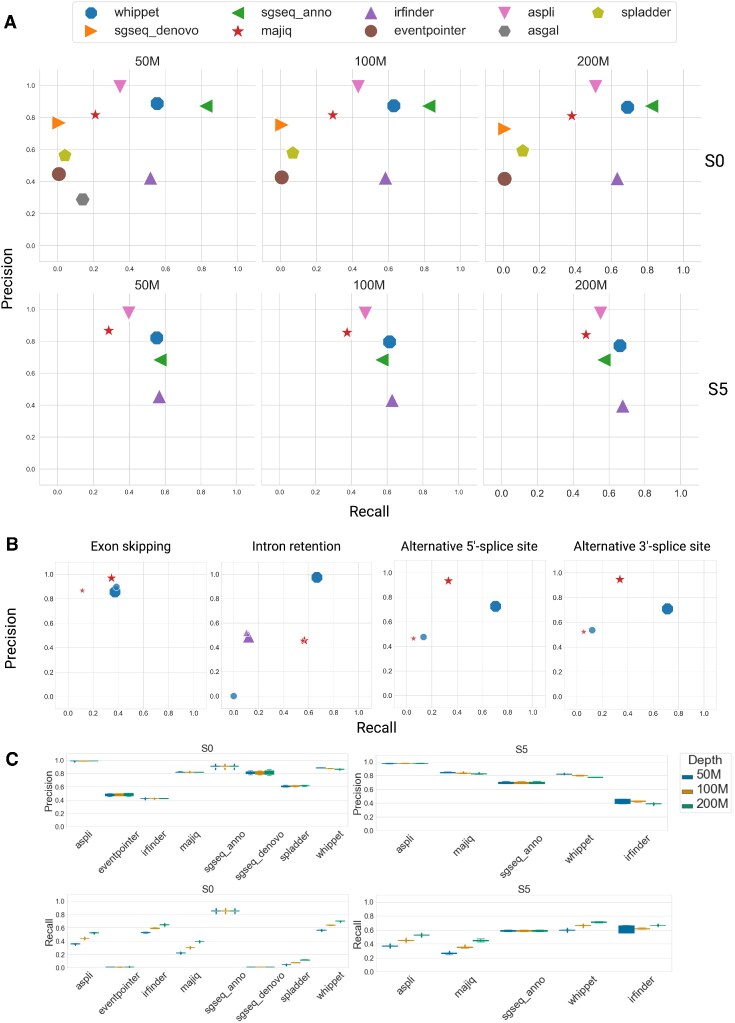
(**A**) Precision/recall plots for AS event detection tools for data sets S1 and S5. The values are taken from the analysis of one out of five simulated datasets as an example. (**B**) Tool performance on S5 (bigger markers) and S5-tr (smaller markers) sets by AS event type for the sequencing depth of 50M reads.(**C**) Precision/recall boxplots for AS event detection tools for the S0 (repeated 5 times) and S5 (repeated 5 times)

Figure 3A (upper part) shows results for data set S0. After examining the results, we excluded EventPointer, SGSeq (*de novo*), and splAdder from the further detailed analysis as these tools demonstrate low recall (less than 10% of recovered events). We also excluded ASGAL because of the long run time (a genome-wide analysis of 50 million reads took around 3 days). Supplementary Figure S4 shows results for data sets S1-S4. The ranking of the tools differs only slightly. Whippet and SGSeq$\_$Anno (annotated transcripts) show the best performance. For all tools in data sets S0-S4, recall depends on the sequencing depth, while precision does not.

MAJIQ was used to derive the proportion of AS event types and combinations from the SHIP cohort (Supplementary Figure S5). We used the proportions as a parameter for ASimulatoR to simulate the biologically-inspired set S5. Note that only the proportion values, but not the detected AS events were used, so MAJIQ and the other tools remained unaware of exact AS events in S5. We used MAJIQ here, since it does not depend on genome annotation and can extract events directly from alignments. The results for data set S5 (Figure 3A, lower part) demonstrate almost the same ranking of the tools.

Additionally, we investigated the ability of tools to detect events *de novo*. For this purpose, we edited a genome annotation file used as input for AS detection tools. Genome annotation describes the exon composition for all transcripts simulated from a gene: main and alternatives. We truncated annotations and kept only the description of the main transcript for each gene. We denote this data set as S5-tr(uncated).

The tested tools differ significantly in their abilities to detect *de novo* events based on truncated genome annotation. ASpli and SGSeq (annotated transcripts) did not detect any novel events. For other tools, we compared precision and recall for S5 and S5-tr for each AS event type individually (Figure 3B). Whippet did not find any *de novo* intron retention events. The recovery rate of alternative splice sites also dropped for all tools: for MAJIQ., precision decreased from around 0.9 to 0.5; recall decreased from 0.4 to 0.1; for Whippet, precision decreased from around 0.7 to 0.4; recall decreased from 0.8 to 0.2 (evaluated for 50M reads).

For data sets S0 and S5 we repeated the AS detection with the full genome annotation five times to evaluate the robustness of the performance. All evaluated AS detection tools demonstrated robust performance (Figure 3C). On data set S5 with the most complex combinations of AS events, the increasing sequencing depth leads to increasing recall but slightly decreasing precision.

**Figure 4.**
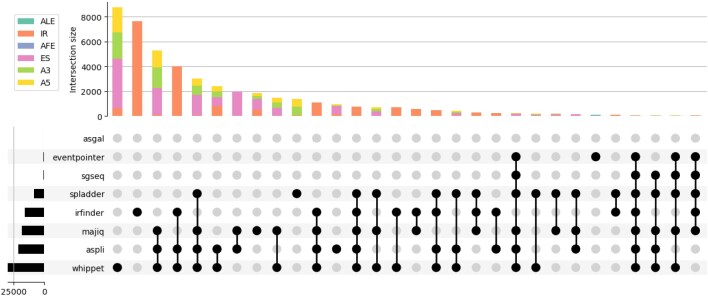
The UpSet plot shows the intersection of results from evaluated AS detection tools.

Notably, the results of different tools show comparably little overlap (Figure 4). Most events are detected exclusively by one tool (e.g., Whippet), suggesting that an integrative approach is needed for the comprehensive analysis of AS events from short-read RNA-Seq data: (i) detect known events using tools based on genome annotation (e.g. ASpli, Whippet); (ii) detect intron retention *de novo* using IRFinder; (iii) detect *de novo* events using Whippet and/or MAJIQ.

### Run time

**Figure 5.**
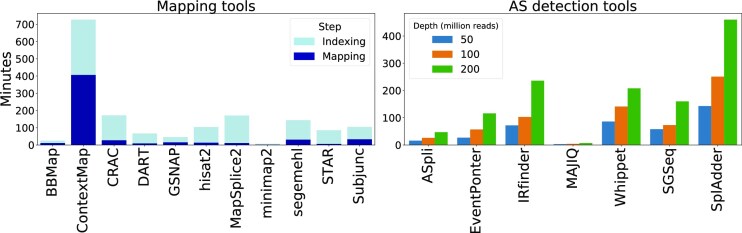
The run time in minutes of the splice-aware mapping tools and AS event detection tools.

Using the data set S0, we estimated the run time of the tools tested on Intel® Xeon® Gold 6148 Processor with 27.5M Cache, 2.40 GHz (Figure 5). For the mapping tools, we used the downsampled data set with 10 million reads from the benchmark. Most tools perform indexing and mapping within an hour. ContextMap is the slowest tool with a run time of around 11 h. minimap2 and BBmap are the fastest tools with a run time of only several minutes. We also estimated the run time of AS detection tools depending on the sequencing depth (Figure 5). The run time of MAJIQ does not depend on the sequencing depth and is in the range of minutes. Most other tools run within several hours, with splAdder taking up to 10 hours for 200 million reads. ASGAL was only evaluated with 50 million reads, already running longer than 72 h to complete.

## DISCUSSION

We investigated the performance of AS event detection tools in a comprehensive benchmark. We started with splice-aware mapping tools to obtain the best possible input for event detection. Among splice-aware mapping tools, STAR and HISAT2 present the best balance between the precision value, fraction of unmapped reads values, and the run time, which is in concordance with previous findings (12).

Concerning AS event detection, we still find much room for improvement since tools with high recall values (Whippet, SGSeq (annotated transcripts), ASpli) can not detect events *de novo*. Vice versa, tools that can identify events *de novo* demonstrate poor recall values. We suggest using a combination of existing tools for a comprehensive AS analysis on the event level using short-read RNA-Seq data.

While we aimed for a comprehensive, objective, and standardized benchmark, we faced some limitations. First, the simulated data sets might not reach the same level of complexity as real biological data sets. We mitigated this by mimicking the proportions of AS event types as observed in the SHIP cohort. Second, for run time reasons, we refrained from a tool-specific parameter tuning and relied on the default parameters, which should ideally lead to optimal results in typical settings. Additionally, we did not want to favor tools with higher flexibility simply due to an increased number of parameters to tune. Finally, we did not explore the performance of the machine and deep learning-based tools (e.g. DARTS (43), IRFinder-S (44)). The simulated AS events were added randomly and do not account for, e.g., regulatory sequences that are used in machine learning approaches. Finally, our benchmark is based on a human genome annotation. The results might differ for organisms with dissimilar AS patterns (e.g. for plants). However, Baruzzo et al. (12)showed that at least the performance of RNA-Seq mapping tools does not differ dramatically between human and *Plasmodium falciparum* genomes.

Many challenges in AS analysis have yet to be addressed, including general difficulties for tool development such as the lack of efficient parallelization and substantial run time. We found that the standard format for aligned reads—bam/sam/cram—differs between tools and might not be compatible with some AS event detection tools. For instance, only few mapping tools add an XS tag that indicates the strand orientation of an intron which is needed by tools such as SGSeq. We gathered the information about tools compatibility here: https://dicast.readthedocs.io/en/master/tools/tools.html. This incompatibility limits the usability of many splice-aware alignment tools for AS analysis. While DICAST introduces a unifying standard for AS event reporting, AS event detection tools utilize inherently different approaches and lead to inconsistent results. To mitigate this, DICAST allows users to execute any combination of tools and facilitates adding newly published tools. By standardizing the output of AS event detection tools, DICAST significantly simplifies downstream analysis. In summary, DICAST offers a unified interface for existing methods and boosts method development by offering an easily extensible framework for benchmarking of existing and novel AS analysis approaches.

## DATA AVAILABILITY

Access to the SHIP data for research purposes may be requested at https://www.fvcm.med.uni-greifswald.de/dd_service/data_use_intro.php. The description of simulated data sets and the corresponding R scripts are available at https://doi.org/10.5281/zenodo.7573144.

## Supplementary Material

lqad044_Supplemental_File
