# Supplementary material for: Alternative splicing analysis benchmark with DICAST
Source: NAR Genom Bioinform. 2023 May 30;5(2):lqad044. doi: 10.1093/nargab/lqad044 (PMC10227362; doi:10.1093/nargab/lqad044)
Supplement: lqad044_Supplemental_File [file lqad044_supplemental_file.pdf]

Supplemental Material
Alternative splicing analysis benchmark with DICAST

Amit Fenn*[,1,2], Olga Tsoy*[,2], Tim Faro[1], Fanny Rössler[1], Alexander Dietrich[1], Johannes Kersting[1], Zakaria Louadi[1,2], Chit Tong Lio[1,2], Uwe Völker[3,4], Jan Baumbach[2,5], Tim Kacprowski[,6,7], and Markus List**[,1,+]**

[1]Chair of Experimental Bioinformatics, Technical University of Munich, 85354 Freising, Germany
[2]Institute of Computational Systems Biology, University of Hamburg, Notkestrasse 9, 22607 Hamburg, Germany
[3]Interfaculty Institute for Genetics and Functional Genomics, University Medicine Greifswald, Felix-Hausdorff-Straße 8, D-17475 Greifswald, Germany;
[4]DZHK (German Centre for Cardiovascular Research), Partner Site Greifswald, Greifswald, Germany
[5]Institute of Mathematics and Computer Science, University of Southern Denmark, Campusvej 55, 5000 Odense, Denmark
[6]Division Data Science in Biomedicine, Peter L. Reichertz Institute for Medical Informatics of Technische Universität Braunschweig and Hannover Medical School, Braunschweig, Germany
[7]Braunschweig Integrated Centre of Systems Biology (BRICS), TU Braunschweig, Braunschweig, Germany
[+]corresponding author: markus.list@tum.de
*joint first authors
**joint last authors

ABSTRACT

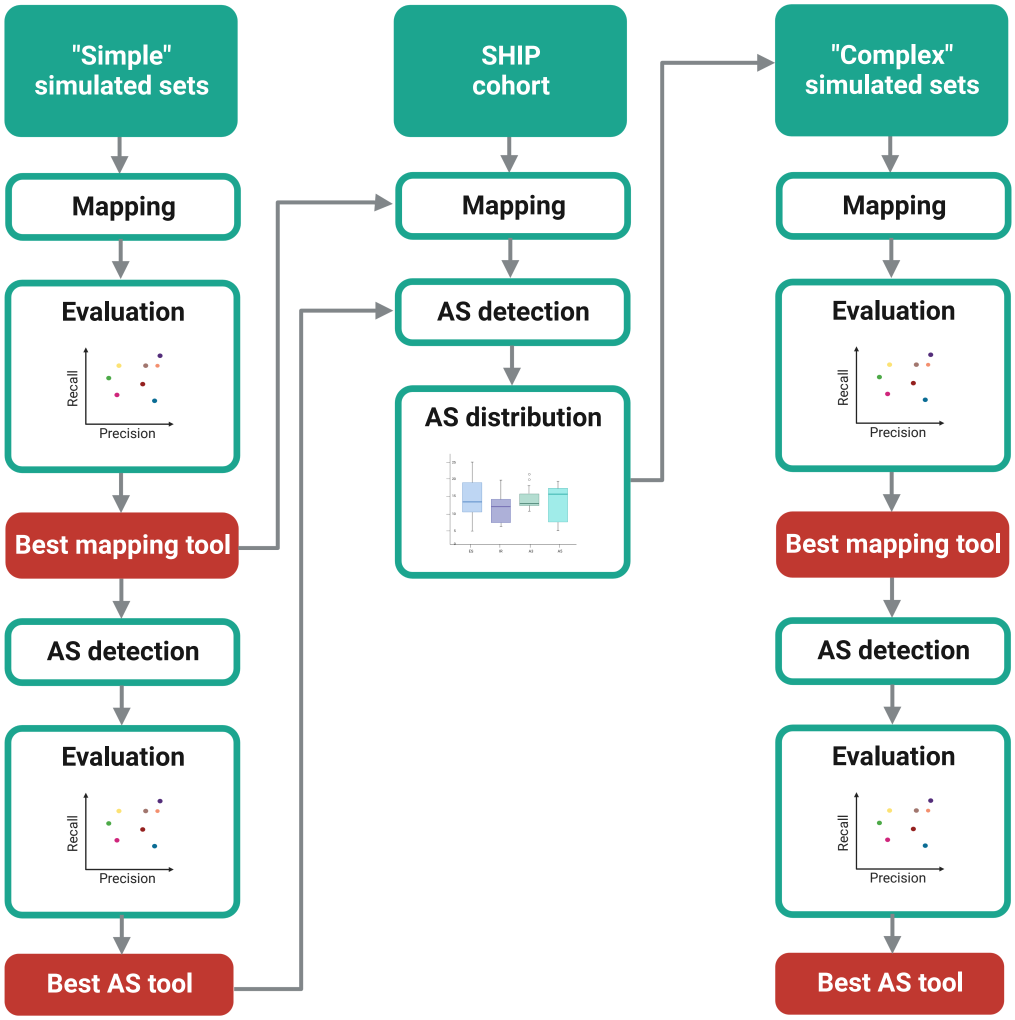

Figure S1. The workflow used to benchmark AS analysis tools. Created with BioRender.com

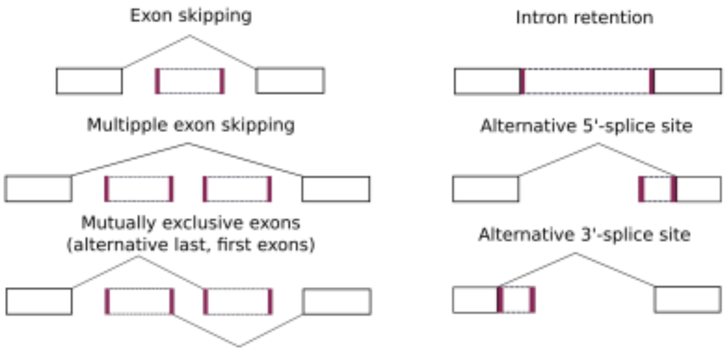

Figure S2. The coordinates of AS events reported in the unified format output of DICAST are shown as bold purple lines in the corresponding splice graph

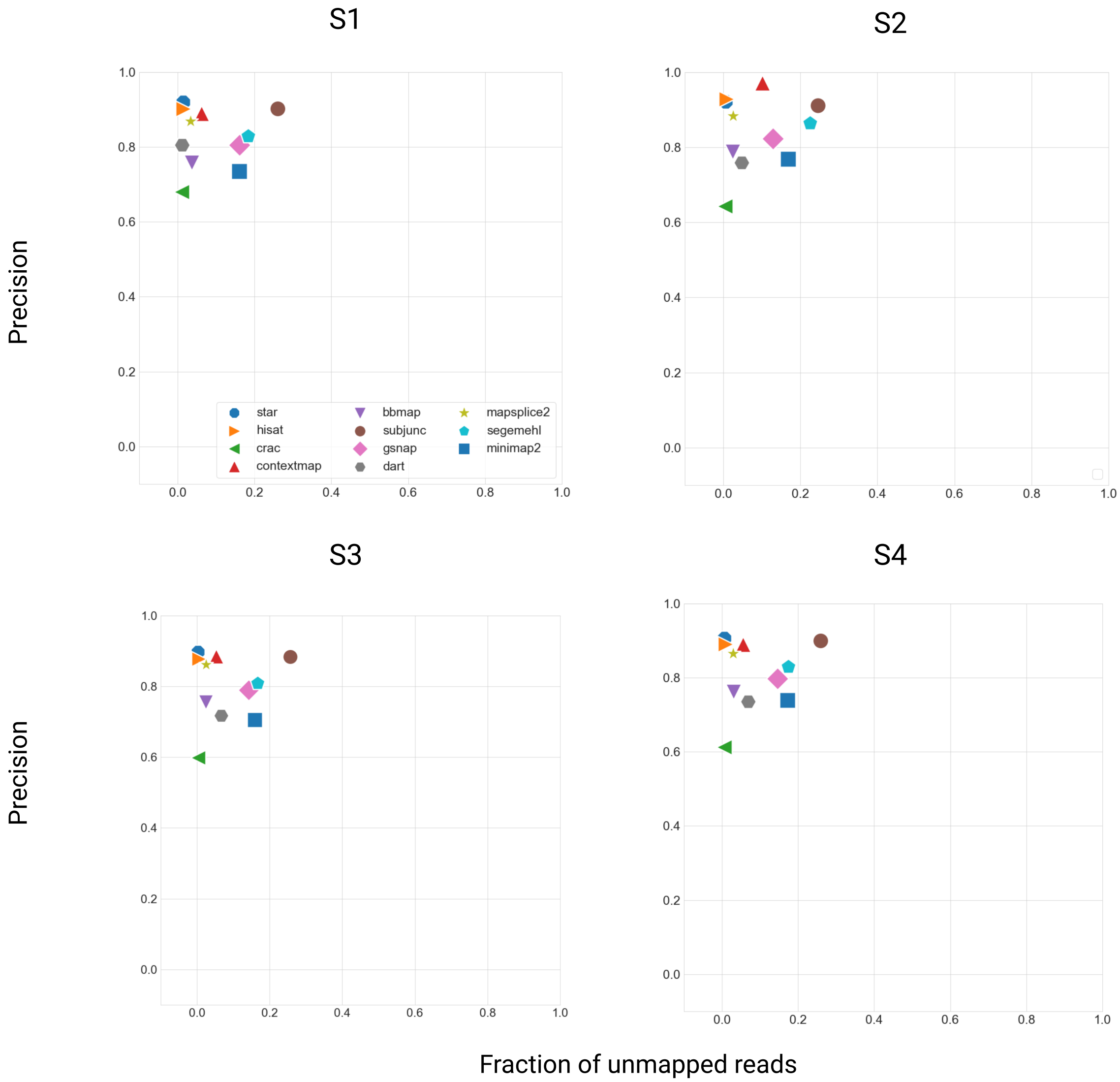

Figure S3. The plots for precision and fraction of unmapped reads for splicing-aware mapping tools for the S1-S4 simulated sets

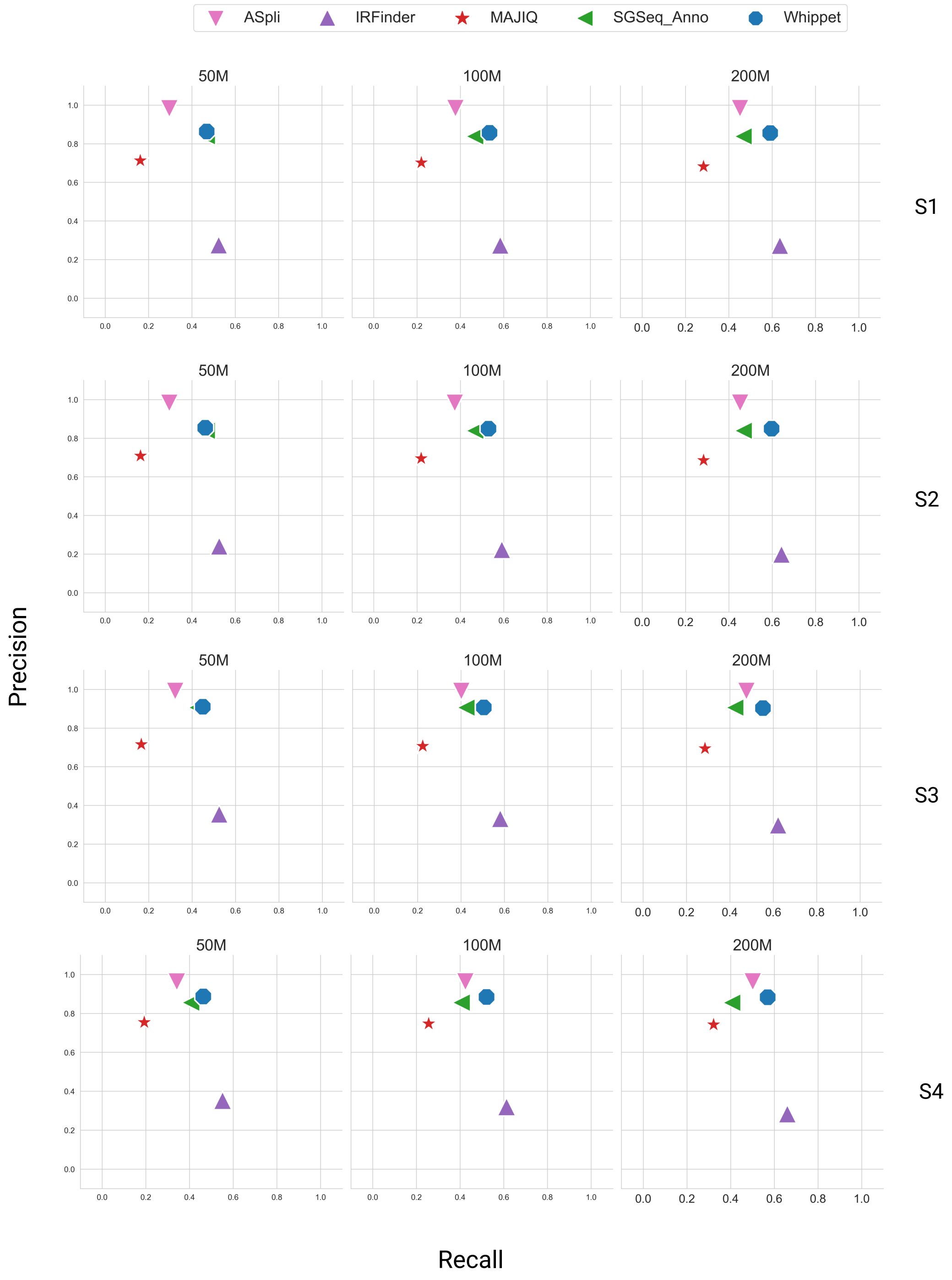

Figure S4. Precision/recall plots for AS event detection tools for the S1-S4 simulated sets

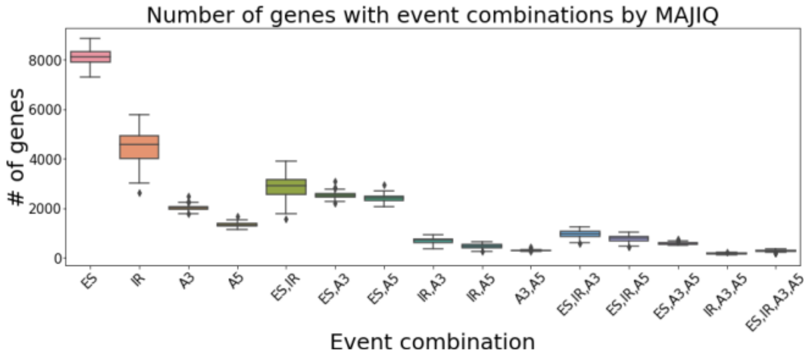

Figure S5. Number of genes with AS event types found by MAJIQ in the SHIP cohort data set
